# Prevalence and molecular characterization of cystic echinococcosis in livestock in the Hazara Division, Pakistan

**DOI:** 10.3389/fvets.2025.1542572

**Published:** 2025-02-24

**Authors:** Safia Arbab, Hanif Ullah, Inam Ul Hassan, Suleman, Weiwei Wang, Abdul Qadeer, Jiya Zhang

**Affiliations:** ^1^Key Laboratory of Veterinary Pharmaceutical Development, Ministry of Agriculture, Lanzhou, China; ^2^Key Laboratory of New Animal Drug Project of Gansu Province, Lanzhou, China; ^3^Lanzhou Institute of Husbandry and Pharmaceutical Sciences, Chinese Academy of Agricultural Sciences, Lanzhou, China; ^4^West China School of Nursing, Sichuan University, Chengdu, China; ^5^Department of Microbiology, Hazara University Manshera, Manshera, Pakistan; ^6^Department of Zoology, Government Post Graduate College, Swabi, Pakistan; ^7^Higher Education Department, Civil Secretariat Peshawar, Peshawar, Pakistan; ^8^Department of Cell Biology, School of Life Sciences, Central South University, Changsha, China

**Keywords:** prevalence, echinococcosis, livestock, molecular characterization, Hazara Division, *Echinococcus granulosus*

## Abstract

**Introduction:**

Echinococcosis is a parasitic zoonotic disease caused by *Echinococcus* tapeworm larvae, forming cysts in organs like the liver and lungs. It primarily affects livestock and humans, with significant public health and economic implications worldwide. In the Hazara Division, the prevalence and genetic diversity of the *Echinococcus* is largely unexplored. Therefore, the current study aims to assess the prevalence o*f Echinococcus granulosis* (EG) in livestock.

**Materials and methods:**

From April 2023 to April 2024, a total of 480 livestock animals, including buffaloes, cattle, goats, and sheep, were examined for echinococcosis across various slaughterhouses in the Hazara Division. Cysts of *Echinococcus* were collected from different organs, and the cyst fluid (CF) was microscopically analyzed before DNA extraction. PCR amplification was performed targeting the Cox1 (317 bp) and Cyto B (309 bp) genes to confirm the presence of *E. granulosus*.

**Results:**

The overall prevalence of Cystic echinococcosis (CE) was 12.2% (59/480), with rates observed in different species as follows: cattle (13.1%), buffaloes (15.2%), goats (6.3%), and sheep (5.7%), (*p*-0.658). District-wise, higher prevalence rates were recorded in Haripur and Mansehra districts (17.5% and 16.2%), followed by Abbottabad and Battagram (12.5% and 11.2%). The lowest prevalence of infection was observed in the upper and lower Kohistan districts, with rates ranging from (8.7% and 7.5%), respectively. The infection was more common in male animals than in females, particularly among those older than 4-5 years (*p* =0.048). Sex-wise prevalence varied across species, with cattle showing rates of 12.4% in males and 14.3% in females. In buffaloes, prevalence was 20% in males and 13.3% in females, followed by sheep at 5% in males and 6.2% in females, and goats at 8% in males. Most animals in the study were older than three years, with the highest number of cysts found in animals over five years of age. Hydatid cysts were most found in the liver (39.1%) and lungs (34.7%), followed by the kidneys (17.3%) and heart (8.6%), (*p*-0.01).

**Conclusion:**

In conclusion, E.G. is highly prevalent in the livestock population of the Hazara division.

## 1 Introduction

Cystic echinococcosis (CE) is a zoonotic disease caused by the larval stage of *Echinococcus granulosus*, a parasitic tapeworm. It is a major One Health concern, as its transmission involves humans, animals, and the environment. The disease spreads through contact with infected animals, particularly dogs, and exposure to contaminated soil, food, or water containing tapeworm eggs. Preventing and controlling CE requires a collaborative approach, integrating public health measures, veterinary interventions, and environmental management strategies (1, 2). It primarily affects the liver and lungs, forming cysts that may cause organ dysfunction if untreated. Both humans and animals can be affected by this parasitic infection, which is typically transmitted through the ingestion of eggs from contaminated food, water, or contact with infected animals. The larvae of *Echinococcus* develop cysts in various organs, causing serious health issues (3). Echinococcus eggs are highly resilient in the environment, surviving for months under suitable conditions. CE involves the formation of large, fluid-filled cysts, mainly in the liver and lungs, causing symptoms such as abdominal pain, respiratory problems, and, in severe cases, cyst rupture leading to anaphylaxis. AE grows invasively like a malignant tumor, predominantly in the liver, with high mortality if untreated due to liver failure or metastasis (4).

Echinococcosis affects more than a million people globally at any given time. CE is common in rural and pastoral areas, while AE is typically found in temperate regions such as Central Asia, China, and parts of Europe. Infected livestock experience a decrease in meat and milk production and reduced hide quality. The economic burden for humans is significant due to the high costs of medical treatment and loss of productivity (5).

CE varies in its characteristics across different geographic regions and host populations, owing to the morphological and biological differences within the *E. granulosus* population (6). Mitochondrial and nuclear genetic markers examine molecular and genotypic variation, with multiple genotypes (G1-G10) identified within *E. granulosus* (7). Prevalence studies and molecular identification are crucial for developing effective control strategies and preventive measures, which can help reduce the economic impact of CE (6). Pakistan is an agrarian country, and over the years, livestock has become the largest subsector of agriculture. Over 8 million rural families rely on agriculture, with 35–40% of their income derived from livestock production (8). Livestock plays a vital role in the gross domestic product (GDP) and economic sustainability of Pakistan. Agriculture contributes around 21% to the national GDP, with livestock accounting for approximately 11.9% of that share (8). Pakistan’s livestock sector has been significantly impacted by CE, leading to substantial economic losses (9).

There are limited studies on the prevalence of *E. granulosus* in Pakistan, and they do not offer enough information about the geographical distribution or the etiological agents of CE (10). This study seeks to evaluate the presence of *E. granulosus* in livestock, including cows, buffaloes, goats, and sheep, in the northern regions of Khyber Pakhtunkhwa, using PCR-based techniques for confirmation. The findings will help establish the prevalence of CE and provide a basis for future research on the disease’s diagnosis, control, and prevention.

## 2 Materials and methods

### 2.1 Ethical statement

The Ministry of Science and Technology of the People’s Republic of China has established regulations to ensure the humane treatment and use of laboratory animals, focusing on minimizing their distress. This research was supported by funding from the National Natural Science Foundation of China and the China Agriculture Research System (CARS-37).

### 2.2 Study area

**Figure 1:**
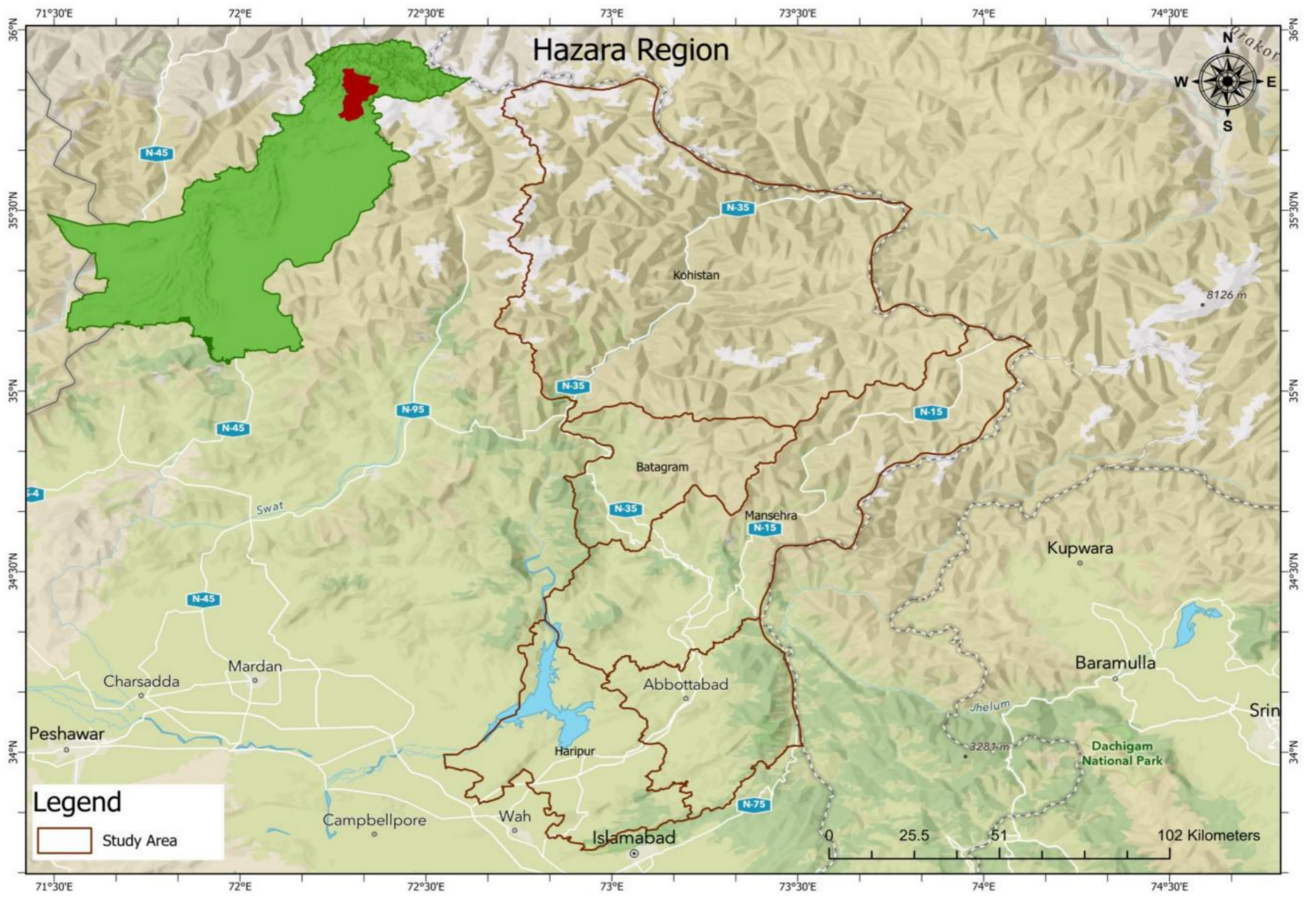
Map showing the study area.

Hazara Division, located in the Khyber Pakhtunkhwa province of Pakistan, lies along the Indus River and includes districts: Abbottabad, Mansehra, Haripur, Battagram, Upper Kohistan, and Lower Kohistan. It shares borders with Gilgit-Baltistan and Azad Jammu and Kashmir to the north and east, Islamabad and Punjab to the south, and Malakand and Mardan Divisions to the west. Renowned for its picturesque landscapes, Hazara Division also holds significant strategic importance due to its location show in Figure 1.

### 2.3 Study duration

From April 2023 to April 2024, a total of 480 animal samples were collected for study, comprising 175 cattle, 190 buffaloes, 52 sheep, and 63 goats. CE were extracted from various organs of the livestock slaughtered in the designated areas of Hazara Division during this period.

### 2.4 Sample collection and storage

The study involved the collection of a total of 480 animal samples, including 175 cattle, 190 buffaloes, 52 sheep, and 63 goats, from various slaughterhouses across Hazara Division. The samples were obtained from animals slaughtered between April 2023 and April 2024. The research was conducted in specific slaughterhouses located in key districts of the division, such as Abbottabad, Mansehra, Haripur, and Battagram, which were identified based on their high livestock throughput. On average, the research team conducted visits to these slaughterhouses twice a month to ensure consistent sample collection throughout the study period. CE were extracted from the organs of the slaughtered animals, which were then analyzed for further investigation.

Each animal underwent an antemortem examination to check for any physical anomalies. During the postmortem assessment, cattle were visually inspected for cysts. If any unusual anatomical features were observed, organ palpation was performed for further examination. The liver, lungs, spleen, muscles, kidneys, and heart organs that commonly contain cysts were collected in sterile, zip-locked polythene bags. Each sample was labeled with a unique ID for proper identification. The collected samples were then stored temporarily at 4°C for preservation (11). CE samples were collected aseptically from slaughtered animals. The samples were transported to the laboratory in cool boxes containing sterile normal saline. This approach allowed for a comprehensive understanding of the prevalence and distribution of CE across different animal species in Hazara Division.

### 2.5 Examination of cysts and assessment of protoscoleces viability

CF was carefully aspirated using a 5-ml syringe under aseptic conditions and transferred into sterile Falcon tubes. The tubes were stored at −4°C until further analysis. The fluid was centrifuged for 8 min at 3,000 rpm, after which the supernatant was discarded, leaving the pellet at the bottom. The pellet was mixed thoroughly, and a single drop was placed on a glass slide for microscopic examination at ×40 magnification to determine cyst fertility or sterility based on the presence or absence of protoscolices.

The cyst’s germinal layer was also examined for brood capsules or protoscolices by placing a piece of the layer on a slide with glycine. Fertility was confirmed if amoeboid-like peristaltic movement (flame cell activity) was observed under the microscope. In the absence of brood capsules or protoscolices in the examined fluid, the cyst was categorized as sterile (12).

### 2.6 DNA extraction from cystic

Following the manufacturer’s protocol, tissue from the cyst germinal layer was processed for genomic DNA extraction using the DNeasy Blood and Tissue Kit (Qiagen, Hilden, Germany) after isolating total nucleic acids from the cyst samples. The DNA concentration was determined using a NanoDrop spectrophotometer (Thermo Fisher Scientific, Wilmington, DE, USA). Before PCR amplification, the DNA was stored at −20°C after being rinsed in 25–30 μL of RNase-free water. The concentration and purity of the DNA were further assessed using the NanoDrop 2000 spectrophotometer (Thermo Fisher Scientific, USA).

### 2.7 Primer designed and PCR amplification

NCBI Primer-BLAST was used to design species-specific primer sets for the identification of EG. For Cox1 gene (317 bp), the forward primer (F) was 5′-TTACGTTG CCTGTTTTGGCTG −3′ and the reverse primer (R) was 5′-AGCCGTCTTCACATCCAACC-3′. For Cyt B, (309 bp) forward primer (F) was 5′-CGGTGTCCGGTGATACGTTA-3′ and the reverse primer (R) was 5′-CCGGCTTAATCCTAACTGGAG-3′ (13, 14). These primer sets were used for PCR amplification. The PCR was performed in a total reaction volume of 20 microliters, consisting of the following components: 10 μL of PCR master mix (10102ES08, 2x Hieff Tm PCR mix), 1 μL of forward primer, 1 μL of reverse primer, 3 μL of template DNA, and 5 μL of distilled deionized water (dd-water). The PCR conditions were set up as follows: a 5-min initial denaturation at 94°C, 35 cycles of denaturation at 94°C for 30 s, annealing at 58°C for 60 s, and extension at 72°C for 60 s, followed by a final extension step at 72°C for 5 min. All PCR reactions were conducted using a PCR system. The PCR products were then analyzed by electrophoresis on a 2.0% agarose gel stained with ethidium bromide. The final amplicons were visualized under UV transillumination according to their fragment sizes after pre-staining with ethidium bromide.

### 2.8 Statistical analysis

Graphical representations were generated using Microsoft Office Excel 2007. Descriptive statistics, including frequency and percentage analysis, were calculated for the study. Epidemiological data for various variables were analyzed using SPSS Statistics. A *p*-value of 0.05 was used as the threshold for statistical significance.

## 3 Results

### 3.1 Prevalence of CE in livestock

A total of 480 slaughtered animals, comprising 175 cattle, 190 buffaloes, 52 sheep, and 63 goats, were studied from April 2023 to April 2024. The overall prevalence of CE was found to be 12.2% (59/480) across all species. Table 1 presents the prevalence of hydatid cysts in different livestock species, categorized by sex. Among cattle, 10.4% of males and 14.2% of females were infected, resulting in an overall infection rate of (13.1%). In buffaloes, the infection rate was higher, with (16.3%) of males and 13% of females affected, yielding a total prevalence of (15.2%). Sheep exhibited the lowest infection prevalence, with 5% of males and 6.2% of females affected, resulting in an overall rate of 5.7%. In comparison, goats had a moderate infection rate, with 8% of males and 5.2% of females infected, giving a total prevalence of 6.3% (*p* = 0.658). This data indicates that buffaloes had the highest prevalence of hydatid cysts, while sheep had the lowest, with male and female infection rates varying slightly within each species.

**Table 1:** The sex wise prevalence of CE in various slaughtered animals.

Species	Male (P/N, %)	Female (P/N, %)	*p*-value
Cattle	13/105 (12.4%)	10/70 (14.3%)	0.658
Buffalo	11/55 (20%)	18/135 (13.3%)	
Sheep	1/20 (5%)	2/32 (6.2%)	
Goat	2/25 (8%)	2/38 (5.3%)	

### 3.2 Prevalence of CE different livestock species

The Table 2 presents the prevalence of CE in different livestock species, showing the total number of animals, the number and percentage of positive cases, and the number and percentage of negative cases. In cattle, 23 out of 175 animals (13.1%) were found to be positive for Cystic Echinococcosis, while 152 animals (86.9%) were negative. In buffalo, 29 out of 190 animals (15.2%) tested positive, and 161 animals (84.8%) were negative. Sheep had the lowest prevalence, with only 3 out of 52 animals (5.7%) testing positive, and 49 animals (94.3%) negative. In goats, 4 out of 63 animals (6.3%) were positive, and 59 animals (93.7%) were negative.

**Table 2:** Shows the prevalence of CE in livestock animals, positive cases, and negative cases with their respective percentages.

Species	Total animals	Positive (N)	Positive (%)	Negative %	Negative (%)
Cattle	175	23	13.1%	152	86.9%
Buffalo	190	29	15.2%	161	84.8%
Sheep	52	3	5.7%	49	94.3%
Goat	63	4	6.3%	59	93.7%

### 3.3 District-wise prevalence

**Figure 2:**
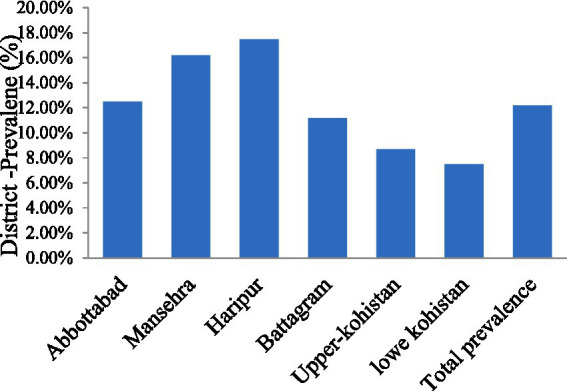
The frequency of CE in the Hazara Division.

Table 3 presents the prevalence of CE in livestock across different districts. In Abbottabad, 10 out of 80 animals (12.5%) were positive, while in Mansehra, 13 out of 80 animals (16.2%) tested positive. Haripur had the highest prevalence with 14 out of 80 animals (17.5%) testing positive, followed by Battagram with 9 out of 80 (11.2%). In Upper Kohistan, 7 out of 80 animals (8.7%) were positive, and Lower Kohistan had 6 out of 80 animals (7.5%) testing positive. Across all districts, a total of 59 positive cases were observed out of 480 animals, resulting in an overall prevalence of (12.2%) as shown in Figure 2.

**Table 3:** Prevalence of CE among different districts of Hazara Division.

District prevalence	Positive	Negative	Total	Prevalence (%)
Abbottabad	10	70	80	(12.5%)
Mansehra	13	67	80	(16.2%)
Haripur	14	66	80	(17.5%)
Battagram	9	71	80	(11.2%)
Upper Kohistan	7	73	80	(8.7%)
Lower Kohistan	6	74	80	(7.5%)
Total	59	421	480	(12.2%)

### 3.4 Characteristics and age/organs wise prevalence of CE

The age-wise distribution of CE revealed a higher prevalence in older animals (over 4–5 years), with both males and females showing relatively elevated infection rates. Among the species studied, cattle exhibited the highest prevalence, ranging from 12.7 to 16.6%, followed closely by buffaloes (15.2 to 15.7%). Sheep had a prevalence ranging from 10 to 16.6%, while goats showed an infection rate of 16.6%. The differences in prevalence were statistically significant (*p* = 0.048) as shown in Table 4.

**Table 4:** Age and organs wise prevalence of cysts echinococcosis isolated from infected livestock animals.

Age-wise prevalence	Characteristics				
	Cattle P/N (%) Buffalo P/N (%) Sheep P/N (%) Goat P/N (%)				*p*-value
3 year	3/35 (8.5%)	2/25 (8%)	2/26 (3.8%)	2/34 (5.8%)	0.048
4 year	11/86 (12.7%)	11/70 (15.7%)	2/20 (10%)	1/23 (4.3%)	
5 year and above	9/54 (16.6%)	16/105 (15.2%)	1/6 (16.6%)	1/6 (16.6%)	
Organ-wise					
Heart	2/23 (8.6%)	2/29 (6.8%)	0/3 (00)	0/4 (–)	0.01
Kidney	4/23 (17.3%)	3/29 (10.3%)	0/3 (00)	0/4 (%)	
Liver	9/23 (39.1%)	6/29 (20.6%)	1/3 (33.3%)	1/4 (25%)	
Lungs	8/23 (34.7.0%)	15/29 (17.2%)	1/3 (33.3%)	2/4 (25%)	

Regarding organ susceptibility, the lungs and liver were the most affected, with prevalence rates ranging from 34.7 to 39.1%, followed by the kidneys (17.3%) and heart (8.6%), (*p*-0.01) (Table 4). Histopathological examination of hydrated cysts revealed thickened hepatocyte tissue and fibrous wall proliferation around the cysts in the liver, while the lungs showed thickened bronchiolar and alveolar walls. The distribution of CE across various organs demonstrated significant variations in infection rates.

Microscopic analysis of CE showed that 24/59 samples (40.70%) were sterile, while 35/59 (59.3%) were fertile. Histopathological analysis of hydatid cysts in the liver revealed notable structural changes, including significant thickening of hepatocyte tissue, which is indicative of inflammation and cellular stress in response to the presence of the cyst. In the lungs, the observations highlighted thickening of the bronchiolar and alveolar walls, likely resulting from an inflammatory response and tissue remodeling due to the cyst’s growth. These changes point to the extensive pathological impact of HC on affected organs, potentially impairing both hepatic and respiratory functions.

### 3.5 Molecular identification of the CE

**Figure 3:**
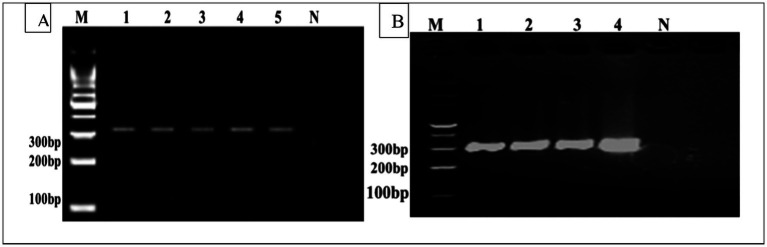
**(A)** M Represents the DNA size marker. Lanes 1–5 correspond to positive samples of the Cox1 gene (317 bp). Lane N represents the negative control. **(B)** Cyto B gene (309 bp), where (M) represents the DNA size marker. Lanes 1–4 correspond to positive samples, and Lane N represents the negative control (not infected).

DNA samples extracted from fertile cysts were amplified using species-specific primers in PCR, producing distinct DNA fragments. PCR amplification produced bands on both Cyto B, 309 bp, and Cox1, 317 bp, respectively. PCR amplification of the mitochondrial Cox1 and Cyt-b genes was successfully confirmed from cyst samples obtained from the liver, lungs, spleen, and mesentery for detection of *E. granulosus* (Figure 3). The PCR analysis established the presence of *E. granulosus* in the six districts.

## 4 Discussion

CE, caused by *E. granulosus*, remains a significant zoonotic disease in many parts of the world, including Pakistan, particularly in rural and agricultural regions. In Hazara Division, the prevalence of CE in livestock animals such as cattle, sheep, and goats has been reported to be relatively high, with studies indicating varying rates of infection across different species. This region, like many others in Pakistan, is characterized by extensive livestock farming, which increases the risk of transmission of the parasite between animals and humans. The molecular characterization of *Echinococcus* species has revealed the existence of multiple genotypes of *E. granulosus* in the region, contributing to the complexity of its epidemiology and transmission dynamics.

CE is a globally prevalent parasitic infection, particularly widespread in Central Asia. Around 270 million people representing 58% of the population in regions such as western China, Iran, Uzbekistan, Afghanistan, Tajikistan, Mongolia, Kazakhstan, Kyrgyzstan, Turkmenistan, and Pakistan are at risk of contracting this disease (15). Despite the global prevalence of both AE and CE, they are still considered neglected zoonoses due to limited awareness and insufficient disease management efforts. While CE is widespread around the world, AE is generally linked to higher pathogenicity and fatality rates, especially in Asia (16). CE is endemic in Pakistan, causing considerable economic losses due to rising healthcare costs and expenses in livestock agriculture.

Furthermore, limited awareness about CE in Pakistan hampers understanding of its transmission dynamics (17, 18). District-wise analysis revealed higher prevalence rates of CE in Mansehra and Haripur districts, ranging from 16.2 to 17.5%, followed by Abbottabad and Battagram with rates between 11.2 and 12.5%. The lowest prevalence was observed in the upper and lower Kohistan regions, where rates ranged from 7.5 to 8.7%. In comparison, reported prevalence rates in slaughtered animals were 0.58% in India, 11.84% in China, and 10.7% in Iran (19–21). The prevalence of CE varies across different regions and provinces of Pakistan. In several parts of Punjab, reported prevalence rates are notably high, with figures reaching 35.0 and 6.67% (22, 23). In contrast, the reported prevalence of CE in Khyber Pakhtunkhwa province was 14.57 and 9.0% in slaughtered animals (8, 11).

In the present study, we observed a prevalence of CE in 480 animals, with infection rates of 13.1% in cattle, 15.2% in buffaloes, 5.7% in goats, and 30% in sheep. Herbivorous animals such as cattle, buffaloes, goats, sheep, and camels serve as intermediate hosts for CE infection (24). A study by Li et al. (20) found a 15.59% prevalence of CE in sheep, followed by a 9.15% positivity rate in cows. Tabar et al. (25) found that the prevalence of CE in buffaloes was significantly higher compared to other species. Our study found that CE was most prevalent in cows (21.7%), buffaloes (17.4%), goats (10.0%), and sheep (9.6%). In contrast, a report from a neighboring region showed the highest incidence in goats (3.25%), sheep (15.38%), cows (15.79%), and buffaloes (15.88%) (11). Ahmed et al. (26) from Balochistan made similar observations, reporting comparable prevalence rates of CE in different animal species. The findings of our study align with numerous other studies, which indicate that cows and buffaloes exhibit a higher prevalence of echinococcosis. This could be due to these animals being more frequently bought, sold, and slaughtered compared to others. In contrast, goats and sheep typically graze in the higher, less contaminated areas of bushes, which are less exposed to echinococcosis eggs, thereby reducing their risk of infection. This grazing behavior may explain the lower prevalence of infection in these species (11).

In the current investigation, the presence of hydrated cysts was attributed to *E. granulosus* infection. The infection was more common in both male and female animals over the age of 4–5 years, with cattle showing the highest prevalence (ranging from 12.7 to 16.6%), followed by buffaloes (15.2 to 15.7%), sheep (10 to 16.6%), and goats (16.6%). Most of the animals studied were older than 3 years, with most cysts found in those older than 5 years. The liver and lungs were the most frequent sites for cyst formation, accounting for 34.7 to 39.1% of cases, while cysts in the kidneys (17.3%) and heart (8.6%) were less common. Histopathological examination of the cysts in the liver revealed thickening of hepatocyte tissue and proliferation of the fibrous wall surrounding the cyst. In the lungs, thickening of the bronchiolar and alveolar walls was also observed. The high prevalence of *E. granulosus* observed in this study aligns with previous reports in humans, where the infection rate was found to be 88.5% (27). In China, the majority of human cases of CE are caused by *E. granulosis*, previously referred to as the G1 strain, which is responsible for around 60% of these cases (28), It is also responsible for 40.62% of the infections reported in India (29). However, there is limited information available on the genetic characterization of *Echinococcus* species in humans in Pakistan. *E. granulosus* has been detected in buffaloes in the Sindh province of Pakistan (30).

According to the organ susceptibility to hydatid cysts, the lungs were found to be more affected (35.3%) than the liver (20.0%), kidneys (2.7%), and heart (1.8%). The distribution of CE among various organs showed significant variation. A recent study from a neighboring area in the same province reported similar findings, with a higher prevalence of cysts in the liver (63.49%), followed by the lungs (23.80%), mesentery (2.64%), and 10.05% of cases involving the heart and kidneys (11). In contrast to our results, the highest percentage of cysts was found in the lungs (47.31%), followed by the liver (25.31%), and the spleen (1.83%). Additionally, a single cyst was observed in both the kidney and heart (31). Consistent with several studies that support our findings, the higher infection rate in the liver may be attributed to its role in receiving digested material first through the hepatic portal vein. This material can contain oncospheres, which have a strong affinity for the liver, leading to a greater prevalence of cysts in this organ (32), The liver typically traps the oncospheres, leading to the formation of cysts. However, under certain circumstances, the oncospheres may escape the liver and migrate to other organs, such as the lungs, heart, kidneys, and brain, where they can also form cysts (33). In the present study, we identified infection with *E. granulosus* in affected animals. This parasite is now recognized as the second most significant cause of CE, following *E. granulosus* itself (27). Globally, *E. canadensis* (G6/7) has been reported in Kenya, as well as in various regions across Africa, Asia, and South America (34); and in many countries in eastern and south-eastern Europe (34). The presence of pig and camel populations in Pakistan creates a potential risk for the spread of *E. canadensis* (G6/7) to human hosts, particularly due to cross-border animal movement from Afghanistan and camel slaughter. The characterization of *E. canadensis* (G6/G7) in humans in Pakistan suggests a connection between the pig, dog and camel-dog transmission cycles. The large pig population in Pakistan poses a significant health risk to people, especially since wild pigs often live near populated areas. Although the camel population in the Punjab province is relatively small, the region’s proximity to Afghanistan and Iran, along with illegal animal trafficking across the border, increases the likelihood of species transmission (35).

CE significantly impacts both human lives and economies, yet limited research has been conducted to understand its epidemiology and management in endemic regions, particularly in Pakistan and Balochistan. To gain a deeper insight into the etiology of CE and develop effective management strategies, further studies are needed to assess its prevalence in humans, domestic animals, and wildlife in these areas. Additionally, the lack of public awareness contributes to an increased risk of transmission to both cattle and humans. This situation poses a threat to both human and livestock populations, potentially facilitating the emergence of new mutations and the spread of novel genotypes.

Future research should focus on expanding the geographical scope of the study to cover a wider range of districts within the Hazara Division. Additionally, further molecular characterization, including genetic sequencing and strain analysis, could provide a better understanding of the genetic variability of *Echinococcus* spp. circulating in the region. Surveillance programs should also be intensified to monitor the dynamics of CE transmission, explore potential risk factors, and investigate the role of environmental and husbandry practices in the spread of the disease. Improved diagnostic methods, particularly for early detection of hydatid cysts, and strategies for controlling *Echinococcus* in livestock and human populations will be essential for reducing the burden of this zoonotic disease in the region.

## 5 Conclusion

This study on the prevalence and molecular characterization of CE in livestock from Hazara Division underscores the significant burden of this zoonotic disease in the region. The findings reveal varying infection rates across different livestock species, with cattle and buffalo showing higher prevalence compared to sheep and goats. The molecular analysis identified *E. granulosus* and *E. multilocularis*, with the latter emerging as a noteworthy concern due to its potential zoonotic impact. The study highlights the critical need for improved control strategies, enhanced animal husbandry practices, and public health awareness to mitigate the spread of CE. Additionally, the high prevalence in older animals suggests prolonged exposure, emphasizing the importance of age-specific monitoring and intervention. Overall, this research calls for a coordinated reduce the prevalence of CE in both livestock and humans, ultimately improving public health and animal welfare in Hazara Division.

## Data Availability

The original contributions presented in the study are included in the article/supplementary material, further inquiries can be directed to the corresponding authors.
